# Precursors of self-reported subclinical hypomania in adolescence: A longitudinal general population study

**DOI:** 10.1371/journal.pone.0253507

**Published:** 2021-06-18

**Authors:** Louise Gunhard Nielsen, Martin Køster Rimvall, Jim Van Os, Frank Verhulst, Charlotte Ulrikka Rask, Anne Mette Skovgaard, Else Marie Olsen, Pia Jeppesen

**Affiliations:** 1 Child and Adolescent Mental Health Centre, Mental Health Services, Capital Region of Denmark, Hellerup, Denmark; 2 Department of Child and Adolescent Psychiatry, Psychiatry Region Zealand, Roskilde, Denmark; 3 Department of Psychiatry, University Medical Centre Utrecht, Brain Center Rudolf Magnus, Utrecht, The Netherlands; 4 Department of Psychiatry and Psychology, Maastricht University Medical Centre, Maastricht, The Netherlands; 5 Department of Psychosis Studies, King’s College London, King’s Health Partners, Institute of Psychiatry, London, United Kingdom; 6 Department of Clinical Medicine, Faculty of Health and Medical Sciences, University of Copenhagen, Copenhagen, Denmark; 7 Department of Child Psychiatry/Psychology, Erasmus University Medical Center, Rotterdam, The Netherlands; 8 Research Unit, Department of Child and Adolescent Psychiatry, Aarhus University Hospital, Aarhus, Denmark; 9 Department of Clinical Medicine, Aarhus University, Aarhus, Denmark; 10 National Institute of Public Health, University of Southern Denmark, Odense, Denmark; 11 Department of Public Health, Faculty of Health and Medical Sciences, University of Copenhagen, Copenhagen, Denmark; 12 Center for Clinical Research and Prevention, the Capital Region of Denmark, Copenhagen, Denmark; University of Toronto, CANADA

## Abstract

Symptoms of subclinical hypomania (SHM) are common in the general population of adolescents and young adults. SHM are most often transient yet might be risk markers of later bipolar disorder. The current study aimed to assess the clinical correlates of SHM at age 11 in the general population, examine the continuity of SHM from age 11-age 16 and explore the clinical precursors of age 16 SHM. As part of the Copenhagen Child Cohort 2000, 1,632 preadolescents participated in the examination of SHM and various clinical correlates at age 11, 893 were re-assessed for SHM at age 16 years. At age 11, SHM, psychotic experiences and depressive symptoms were assessed by semi-structured psychopathological interviews. Furthermore, the participants were diagnostically assessed by the Development and Well-Being Assessment and interviewed about sleep length. At age 16, SHM was assessed by self-report, using the Hypomania Checklist-32. Cannabis use occurring at age 15 or earlier was assessed at age 16. At age 11, SHM was associated with depressive disorders (Relative Risk [RR] = 2.96 [95% CI 1.26–6.96]), interview-based depressive symptoms (RR = 9.22 [5.93–14.34]), neurodevelopmental disorders (RR = 2.94 [1.66–5.20]), psychotic experiences (RR = 4.51 [2.90–7.01]) and insufficient sleep (RR = 2.10 [1.28–3.43]. In the longitudinal analyses, age 16 SHM was preceded by age 11 SHM (RR = 1.89 [1.02–3.49]), psychotic experiences (RR = 2.06, [1.28–3.33]), emotional disorders (RR = 1.77, [1.02–3.09]) and cannabis use (RR = 3.14, [1.93–5.10]), after mutual adjustment and adjustment for sex, and sociodemographic factors. In conclusion, age 11 SHM was statistically significantly associated with other types of psychopathology in cross-sectional analyses and showed some continuity with later self-reported SHM at age 16. Particularly early psychotic experiences and cannabis use stood out as independent precursors of self-reported SHM and might constitute important risk markers for the development of future SHM and bipolar disorder. An important potential caveat of the current study includes the self-report assessment of SHM.

## Introduction

Mania is characterized by a variety of symptoms including irritability, overactivity, reduced need to sleep and pressured speech [1]. Hypomania is characterized by fewer and less severe symptoms as compared to mania [2]. Mania and hypomania are hallmark symptoms of bipolar disorder (BD) [2], yet further down the severity spectrum, symptoms of subclinical or subthreshold hypomania (SHM) are common in the general population [3, 4]. In the general population, SHM often occurs in adolescence and is primarily a transient phenomenon [5–7]. Nevertheless, SHM has shown some continuity in youths from age 11 to age 16 in a general population study [8]. Moreover, among adults in the general population, SHM precede later mood disorder with high predictive values [9, 10]. Also, SHM in adolescence has been found to predict BD in young adulthood in a dose-response relationship, i.e. higher hypomania score on a continuous scale resulted in higher risk of BD [11].

Other potential risk factors of developing later clinical mania and BD have been examined in the general population. Given that depression is part of BD, unipolar depression (i.e. episodes of depression in the absence of periods with manic/hypomanic symptoms) as a precursor of later BD has been studied rather extensively, and a recent review found that depressive disorder in adolescence is a strong predictor of later BD [12]. Additionally, the review found that depressive episodes in adolescence were particularly strongly associated with later BD in the context of a genetic risk for BD [12]. In a German general population study, depressive- and hypomanic/manic episodes showed bidirectional influences from late adolescence into early adulthood [6]. Moreover, persistence of depressive symptoms in adolescents and young adults from the general population have been found to mark a risk of developing hypomanic/manic episodes 10 years later [5]. However, a recent community-based study did not find emotional difficulties in childhood to be a strong risk factor related to developing SHM in young adulthood [13].

Other domains of psychopathology have also been found to be associated with hypomania. Particularly irritability is a frequent symptom in a broad spectrum of common mental disorders among children and adolescents, e.g. attention deficit hyperactivity disorder (ADHD), depression and autism spectrum disorders [14]. This might complicate differential diagnosis and identification of mania and bipolar disorder in younger age groups [14, 15]. Mixed results have been reported regarding ADHD in adolescence and later development of hypomania. ADHD, oppositional-defiant disorder and conduct disorder in combination in adolescence have been found to increase the risk notably of developing hypomania as a young adult [16]. However, ADHD in adolescence was only weakly associated with the development of hypomania in young adulthood in a British general population study [13]. This is supported by other community-based studies, reporting that ADHD in adolescence was not an important precursor of later development of mania [17, 18].

Mania can be viewed as part of psychosis spectrum psychopathology [19, 20] and genetic- and environmental risk factors of BD and psychotic disorders have been found to overlap [21, 22]. Like hypomania, psychosis can also be viewed on a continuum ranging from subclinical psychotic experiences that are common in the general population, to manifest psychotic disorders at the severe end [23, 24]. Several community studies have found associations between psychotic experiences and hypomania [4, 20, 25, 26], and psychotic experiences have been shown to precede hypomania [26]. Additionally, a bidirectional association between psychotic experiences and hypomania has been suggested [4, 20, 26]. Also, subclinical psychosis in adults with hypomania has shown to be substantially more predictive regarding the development of BD than hypomania alone [4].

Furthermore, a recent review including community-based studies reported a significant positive correlation between self-reported insomnia and hypomania [27]. However, another community-based study found no differences in the duration of sleep among adults with hypomania compared to adults without hypomania [28]. Lastly, cannabis use in adolescence and adulthood has been shown to be associated with an increased risk of later hypomania and mania [18, 29, 30]. However, the possibility that cannabis use could be used as self-medication and thereby be associated with later development of mania, i.e. the self-medication hypothesis, found no support in a study by Henquet et al. who found that mania at baseline, did not predict later cannabis use [29].

Risk factors of hypomania have mainly been explored in late adolescence and adulthood, and research data on SHM in early adolescence are scarce. In order to possibly prevent later development of hypomania among young adults, it is important to identify the early risk factors of SHM. The current study therefore aimed to describe the clinical correlates of SHM in early adolescence, at age 11 years, and examine preadolescent precursors of SHM at age 16 years.

Our main hypothesis was that clinician-assessed SHM at age 11 would predict self-reported SHM at age 16. Moreover, we hypothesized that other putative risk factors of hypomania–including depressive symptoms, emotional disorders, neurodevelopmental disorders, psychotic experiences, reduced duration of sleep and cannabis use would predict self-reported SHM at age 16.

## Methods

### Study population

The Copenhagen Child Cohort (CCC2000) is a birth cohort comprising 6,090 children born in 16 municipalities in the county of Copenhagen in year 2000. The cohort is representative for the children born in Denmark that year regarding key perinatal and sociodemographic characteristic, except more immigrant families at baseline [31]. For the current study, we used data from the 11-year follow-up (data collection from May 2011 through October 2012) and the 16-year follow up (data collection from August 2016 through November 2017). A total of 1,632 children participated in face-to-face clinical interviews including assessment of hypomania at the 11-year follow-up. Attrition analyses regarding the 1,632 children compared with the total sample have been reported in detail elsewhere [32]. Of the 1,632 participating at age 11, a total of 893 (55%) participated in the 16-year-follow-up, at which they completed a self-reported hypomania questionnaire in conjunction with face-to-face examinations. Non-participants at both follow-ups were characterized by more perinatal and sociodemographic adversities, including more immigrant families [31].

### Ethics

The study was approved by the Danish Data Protection Agency (CSU-FCFS-2016-004, I-Suite 04544), and the Local Committee on Health Research Ethics (protocol 16023242). Participation was voluntary. At the 11-year follow-up, the parents gave oral informed consent to the study participation. The written information given to the participants stated that the participation in the study is voluntary, that by answering the questionnaires consent was given and consent can be withdrawn at any time. Separate written consent was not required by the ethical committee [33]. The parents and youths at age 16 received oral information and the youths gave written consent. The data was kept confidentially, was pseudonymized and analyzed on the secure platform of Statistics Denmark. Small cell-sizes below three individuals were not reported in adherence with the ethical requirements of Statistics Denmark to secure participant anonymity.

### Subclinical hypomania assessment at age 11

SHM was assessed by semi-structured face-to-face interviews using the Kiddie Schedules of Affective Disorders and Schizophrenia–Present and Lifetime Version (K-SADS-PL) [34]. SHM was assessed by items from the affective part of the screening section of K-SADS-PL. All items were scored dichotomously as “likely” or “definitely” present vs. “not present” for the last month and lifetime before. In our analyses we defined SHM as having at least one symptom in at least one of the two time periods. Subclinical hypomania symptoms included questions on elation/expansive mood, decreased need for sleep, racing thoughts and increase in goal-directed activity.

The interviews were performed by trained clinicians who underwent training in performing Kiddie-SADS interviews and were supervised in conjunction with video-taped joint ratings approximately every two weeks, supervised by the senior author (PJ) [35].

### Clinical correlates of subclinical hypomania, assessed at age 11

Depressive symptoms and psychotic experiences were also assessed using the semi-structured interview K-SADS-PL [34]. Depressive symptoms were assessed by items from the affective part of the screening section of K-SADS-PL, including questions on depressed mood, irritability and anhedonia. Psychotic experiences were assessed by screening of 22 items of hallucinations, delusion and subjective thoughts disturbances from the psychosis screening interview and psychosis supplement of the Kiddie-SADS.

Sleep duration was assessed by a structured interview. The interview contained questions regarding both sleep onset, awakening time and a number of sleep problems including difficulties falling asleep, nightmares among others [35]. Only sleep duration was included in our study. The cut-off for insufficient sleep was at ≤ 8.5 hours for weekdays, in line with a previous CCC2000 study [35].

### Diagnostic assessment of mental disorders

ICD-10 mental disorders at age 11 were assessed by child and adolescent psychiatrists using the Development and Well-Being Assessment (DAWBA). The DAWBA is an extensive psychiatric interview regarding children aged 5–16 years, their parents and teachers encompassing psychopathology broadly. The DAWBA includes structured questions, which cover the operational diagnostic criteria for both DSM-IV diagnoses and ICD-10 diagnoses, and includes open-ended questions in the end of each section [36]. The questionnaires were accessed online using individual personalized logins for each respondent [35].

Each child was evaluated independently by two child and adolescent psychiatrists (in pairs), and consensus ratings involving the whole group of raters were made in cases of disagreement. The inter-rater reliability was examined for the assessment of diagnoses by three senior child and adolescent psychiatrists, and a good inter-rater reliability was found with a kappa-value of 0.81 [35].

The DAWBA-based ICD-10 diagnoses were divided into two diagnostic groups for the purpose of the current study: emotional disorders (including anxiety disorders, depression and obsessive/compulsive disorders) and neurodevelopmental disorders (including autism spectrum disorders, tics-disorders, oppositional defiant disorder/conduct disorders and ADHD). Depression and ADHD were also considered separately.

### Cannabis use at age 15 or prior, assessed at age 16

Cannabis use was assessed by self-report at the 16-year follow-up, using the World Health Organization’s “Alcohol, Smoking and Substance Involvement Screening test” (ASSIST), complemented with questions on age of first use [37]. To examine cannabis use prior to the assessment of hypomania outcome, we asked the young individual to recall cannabis use occurring at age 15 years or earlier. The variable was dichotomized as not present (never or only once) vs. present (at least twice).

### Subclinical hypomania assessment at age 16

The Hypomania Checklist 32 (HCL-32) is a non-diagnostic self-reported questionnaire on lifetime hypomania symptoms. The HCL-32 was completed during the face-to-face assessment of the 16-year follow-up. The questionnaire contains 32 symptoms, and the participants had to answer “no” or “yes” (i.e. score 0 or 1) [38]. The HCL was originally developed for adults but has also been utilized in a number of studies including adolescents [39–41]. Several studies examining the HCL-32 has found an acceptable internal consistency [41, 42]. In the current study Chronbach’s alpha was high (0.94). The respondents were subjected to a short introductory text prior to responding to the HCL-32. The text described a phase of hypomania as a discrete period with a significant increase in both levels of energy, initiative and change in mood as compared to the responder’s normal state. A large proportion of the sample endorsed 0 of the 32 items. A well-established cut-point on the HCL is not available. We chose a top 10^th^ percentile of sum-scores on the HCL as our measure of SHM, which corresponded to endorsing at least 20 out of 32 symptoms as present. See S1 Appendix for a distribution of the scores.

### Sociodemographic and perinatal risk factors

For adjustment purposes and for attrition analyses, we used register-based data from the Integrated database for Labor Market Research to describe sociodemographic characteristics [43], and data from the Medical Birth Register to describe birth characteristics [44]. We used the Danish Civil Registration System, to link the data of participants with register data [45].

We used a family adversity index for adjustment purposes. The index comprises 6 variables, each scored 0 (not present) or 1 (present), resulting in index scores ranging from of 0–6. The categories are as follows: 1) both parents born outside of Denmark 2) parents not living together at child’s birth 3) any change in family composition over the period 2000–2010 4) young mother (age< 21 years at childbirth) 5) low education of mother in 2010 (≤ 10 years of primary school) 6) household income within the lowest quartile in 2009–2010. The index has been used in previous CCC2000 studies [32].

## Statistics

The statistical analyses were performed using STATA version 15.1. The statistical tests had a significance level of 5% and were 2 sided.

We used chi-square tests for categorical variables to compare the participants with the non-participants still alive at the 11-year follow-up with regard to sociodemographic and perinatal characteristics.

For cross-sectional analyses at age 11, presence/non-presence of SHM was used as the dependent variable. The independent variables at age 11 years were emotional disorders (including depressive disorders which were also viewed separately), neurodevelopmental disorders (including ADHD which was also viewed separately), interview-based depressive symptoms, psychotic experiences, insufficient sleep. We used binary regression analyses with a log-link function to calculate the relative risk (RR) of having hypomania.

For the longitudinal analyses we also used binary regression with a log-link function to examine the associations between SHM and clinical correlates of SHM at age 11 and presence/non-presence of SHM top 10% score at age 16. This was first done for all putative risk factors individually i.e., SHM, emotional disorders, neurodevelopmental disorders, psychotic experiences, reduced duration of sleep and depressive symptoms at age 11 years and cannabis use at age 15 years or prior. The diagnosis of ADHD and depressive disorder at age 11 years were not viewed individually in the longitudinal analyses due to too small cell sizes, in order to adhere to the ethical guidelines described previously.

Subsequently, all risk factors, besides interview-based depressive symptoms, were included in a multivariate binary regression model for mutual adjustment. Interview-based depressive symptoms were not included because they tapped into the same symptoms as depressive disorders within the emotional disorder variable. The Variance Inflation Factor (VIF-value) value was calculated for the multivariate binary regression in order to check for multicollinearity. All the above crude estimates were reported, and subsequently the models were adjusted for sex and the family adversity index.

Lastly, sensitivities analyses were conducted. The associations between age 11 variables and subclinical hypomania at age 16 were also calculated when viewing age 16 hypomania on a continuous scale. This was done for both the univariate and multivariate analyses by use of linear regression. Because the data on hypomania at age 16 were not normally distributed, we used a non-parametric boot-strapping method with 5000 repetitions to estimate 95% bias corrected and accelerated bootstrap confidence intervals.

Further sensitivity analyses were conducted: two multivariate analyses were conducted without including SHM and emotional disorders respectively. Thus, the sensitivity analyses included only one of the two opposite poles (depression and hypomania) of bipolar disorder in the same analysis to avoid over-adjustment for markers of bipolar disorder.

## Results

### Sample characteristic

As described in the method section, attrition analysis for the cross-sectional study population at age 11 years has been reported previously, showing lower frequencies of psychosocial and perinatal adversities among participants compared to non-participants [32]. For the subsample with longitudinal data, participant characteristics were described by comparing the 893 participants with the 739 non-participants (i.e. the adolescents who participated in the 11-year follow-up, but not in the 16-year follow-up). The non-participants differed from the participants regarding sex (more boys), lower maternal age, lower maternal level of education, and a higher number of immigrant parents (Table 1).

**Table 1:** Attrition analysis—characteristics of the participants compared to the non-participants.

	Participants	Non-participants	P-value (Missing)
	(Follow-up study population)	(Dropouts between the 11-year and 16-year follow-up)	
	(N = 893)	(N = 739)	
	N (%)	N (%)	
**Sex (girls)**	488 (54.05)	361 (48.85)	0.02 (0)
**Singleton birth**	862 (96.64)	699 (94.94)	0.07 (3)
**Birth weight**			
**<2500 g**	37 (4.25)	30 (4.16)	
**2500–4499 g**	798 (91.61)	659 (91.27)	0.91 (39)
**> 4500 g**	36 (4.13)	33 (4.57)	
**Maternal age at birth**			
**16–20**	6 (0.67)	15 (2.04)	
**21–30**	406 (45.52)	376 (51.02)	<0.01 (3)
**31–40**	463 (51.41)	338 (45.86)	
**41–46**	17 (1.91)	8 (1.09)	
**Parents born outside Denmark**			
**2 parents**	57 (6.43)	98 (13.48)	
**1 parent**	89 (10.03)	75 (10.32)	<0.01 (18)
**0 parents**	741 (83.54)	554 (76.20)	
**Mother’s completed education by 2010**			
**1–10 years**	74 (8.39)	97 (13.34)	
**11–14 years**	431 (48.87)	356 (48.97)	<0.01 (23)
**15+ years**	377 (42.74)	274 (37.69)	
**Family constitution at childbirth**			
**Both parents**	796 (89.64)	655 (88.75)	
**One parent**	34 (3.82)	29 (3.93)	0.93 (3)
**Reconstituted or child outside family**	61 (6.85)	54 (7.32)	
**Change in family composition by 2010**	195 (21.95)	171 (23.20)	0.55 (7)

### The prevalence of subclinical hypomania in the cross-sectional- and in the follow-up study population

Among the 1,632 adolescents assessed at age 11 years, 82 (5.0%) had experienced at least one subclinical lifetime SHM symptom according to the semi-structured interviews. A total of 91 adolescents out of 893 adolescents (10.2%) had SHM (a-priori cut-off set at 10%) at the follow-up at age 16 years. See Table 2.

**Table 2:** Distribution of youth sex, mental health symptoms and diagnoses at age 11 by lifetime presence of subclinical hypomania (SHM) at age 11 and 16 years.

	*Subclinical hypomania at age 11*			*Subclinical hypomania at age 16*		
	*(cross-sectional study population)*			*(follow-up study population)*		
	*N = 1*.*632*			*N = 893*		
	*Children without hypomania N = 1550*	*Children with hypomania N = 82*	*Missing*	*Adolescents without hypomania N = 802*	*Adolescents with hypomania N = 91*	*Missing*
Symptom/ diagnoses at age 11	n (%)	n (%)	n	n (%)	n (%)	n
*Sex (female)*	803 (51.8)	46 (56.1)	0	436 (54.4)	52 (57.1)	0
Emotional disorders* (ICD 10 diagnosis)	126 (8.2)	11 (13.4)	10	61 (7.6)	12 (13.3)	4
• Depressive disorder	30 (2.0)	7 (8.54)	10	16 (2.0)	<3	4
Interview-based depressive symptoms	203 (13.1)	52 (63.4)	0	127 (15.8)	19 (20.9)	0
Neurodevelopmental disorders** (ICD 10 diagnosis)	97 (6.3)	13 (15.9)	10	49 (6.1)	5 (5.6)	4
• Attention Deficit Hyperactivity Disorder	40 (2.6)	5 (6.1)	10	17 (2.1)	<3	4
Psychotic experiences	144 (9.3)	28 (34.2)	0	71 (8.9)	19 (20.9)	0
Insufficient sleep (≤8.5 hours)	215 (14.0)	20 (24.7)	14	111 (13.9)	15 (16.5)	4
Subclinical hypomania	Not applicable	Not applicable	Not applicable	37 (4.6)	9 (9.9)	0
Cannabis use***	Not applicable	Not applicable	Not applicable	38 (4.9)	14 (16.1)	27

* Emotional disorders include depressive disorders, anxiety disorders and OCD

** Neurodevelopmental disorders include ADHD, conduct disorders, autism spectrum disorders and tics

***Cannabis use is measured as cannabis use at age 15 or prior, used more than once, and based on self-reported data obtained at the 16-year follow-up.

### Cross-section analysis of the clinical correlates of subclinical hypomania at age 11 years

Table 3 shows the associations between having different symptoms and diagnoses at age 11 years and having SHM at age 11 years. Among the clinical correlates, high RRs were found for depressive symptoms (RR = 9.36, 95% CI = 6.09–14.37), psychotic experiences (RR = 4.40, 95% CI = 2.87–6.75), depressive disorders (RR = 4.00, 95% CI = 1.98–8.07) and neurodevelopmental disorders (RR = 2.59, 95% CI = 1.48–4.53). After adjustment for sex and sociodemographic factors, the results did not change markedly. Notably, the broader emotional disorders variable, which mostly included individuals with anxiety disorders, was not statistically significantly associated with SHM (RR = 1.68, 95% CI = 0.91–3.09).

**Table 3:** The association between symptoms/ diagnoses at age 11 and subclinical hypomania at age 11.

SYMPTOMS/	SUBCLINICAL HYPOMANIA AT AGE 11		
DIAGNOSES AT AGE 11	CROSS-SECTIONAL STUDY POPULATION = 1.632		
	*Crude associations*	Adjusted for sex and sociodemographic factors***	*Missing*
	Relative Risk (95% CI)	Relative Risk (95% CI)	n
EMOTIONAL DISORDERS* (ICD-10 DIAGNOSIS)	1.68 (0.91–3.09)	1.37 (0.70–2.70)	10
• DEPRESSIVE DISORDER	4.00 (1.98–8.07)	2.96 (1.26–6.96)	10
INTERVIEW-BASED DEPRESSIVE SYMPTOMS	9.36 (6.09–14.37)	9.22 (5.93–14.34)	0
NEURODEVELOPMENTAL DISORDERS** (ICD-10 DIAGNOSIS)	2.59 (1.48–4.53)	2.94 (1.66–5.20)	10
• ATTENTION DEFICIT HYPERACTIVITY DISORDER	2.28 (0.97–5.35)	2.63 (1.11–6.24)	10
PSYCHOTIC EXPERIENCES	4.40 (2.87–6.75)	4.51 (2.90–7.01)	0
INSUFFICIENT SLEEP (≤8.5 HOURS)	1.93 (1.19–3.14)	2.10 (1.28–3.43)	14

* Emotional disorders include depressive disorders, anxiety disorders and OCD

** Neurodevelopmental disorders include ADHD, conduct disorders, autism spectrum disorders and tics

****Missing data on 59 individuals on sociodemographic factors.

### Longitudinal analyses

Table 4 shows the bivariate analyses. SHM at age 11 years showed some continuity with self-reported SHM at age 16 years (RR = 2.02, 95% CI = 1.09–3.76). We also found longitudinal associations between psychotic experiences (RR = 2.35, 95% CI = 1.49–3.71), emotional disorders (RR = 1.72 95% CI 0.98–3.01, statistically non-significant) and cannabis use (RR = 3.00, 95% CI = 1.82–4.94) and later self-reported SHM at age 16. Neurodevelopmental disorders, insufficient sleep, and the interview-based measure of depressive symptoms were not longitudinally associated with adolescent SHM. The risk ratios did not change substantially after adjusting for sex and sociodemographic factors.

**Table 4:** The association between symptoms/ diagnoses at age 11 and subclinical hypomania at age 16.

SYMPTOMS/	SUBCLINICAL HYPOMANIA AT AGE 16		
DIAGNOSES AT AGE 11	FOLLOW-UP STUDY POPULATION = 893		
	*Crude analyses*	Adjusted for sex and sociodemographic factors****	*Missing*
	Relative Risk (95% CI)	Relative Risk (95% CI)	n
SUBCLINICAL HYPOMANIA	2.02 (1.09–3.76)	2.05 (1.10–3.80)	0
EMOTIONAL DISORDERS* (ICD-10 DIAGNOSIS)	1.72 (0.98–3.01)	1.71 (0.97–3.00)	4
INTERVIEW-BASED DEPRESSIVE SYMPTOMS	1.35 (0.84–2.17)	1.34 (0.84–2.17)	0
NEURODEVELOPMENTAL DISORDERS** (ICD-10 DIAGNOSIS)	0.91 (0.39–2.15)	0.90 (0.38–2.14)	4
PSYCHOTIC EXPERIENCES	2.35 (1.49–3.71)	2.38 (1.51–3.77)	0
INSUFFICIENT SLEEP (≤8.5 HOURS)	1.20 (0.71–2.01)	1.24 (0.73–2.08)	4
CANNABIS USE***	3.00 (1.82–4.94)	3.21 (1.95–5.28)	27

* Emotional disorders include depression, anxiety and OCD

** Neurodevelopmental disorders include ADHD, conduct disorder, autism spectrum disorder and tics

***Cannabis use is measured as cannabis use at age 15 prior and used more than one time. It is based on self-reported data obtained at the 16-year follow-up.

****Missing data on 25 individuals on sociodemographic factors.

In multivariate analyses, after mutual adjustment and adjustment for sociodemographic adversities, the risk ratios for all the symptoms and diagnoses were largely unchanged (see Table 5).

**Table 5:** Multivariate analysis–the association between symptoms/ diagnoses at age 11 and subclinical hypomania at age 16.

SYMPTOMS/	SUBCLINICAL HYPOMANIA AT AGE 16	
DIAGNOSES AT AGE 11	FOLLOW-UP STUDY POPULATION, N = 893	
	*Crude analyses*	Adjusted for sex (1 = girls) and socioeconomic factors****
	Relative Risk (95% CI)	Relative Risk (95% CI)
SUBCLINICAL HYPOMANIA	1.81 (0.96–3.44)	1.89 (1.02–3.49)
EMOTIONAL DISORDERS* (ICD-10 DIAGNOSIS)	1.72 (0.97–3.07)	1.77 (1.02–3.09)
NEURODEVELOPMENTAL DISORDERS** (ICD-10 DIAGNOSIS)	0.42 (0.16–1.10)	0.41 (0.16–1.03)
PSYCHOTIC EXPERIENCES	1.98 (1.21–3.24)	2.06 (1.28–3.33)
INSUFFICIENT SLEEP (≤8.5 HOURS)	1.07 (0.64–1.79)	1.20 (0.73–1.97)
CANNABIS USE***	2.80 (1.69–4.65)	3.14 (1.93–5.10)

* Emotional disorders include depression, anxiety and OCD

** Neurodevelopmental disorders include ADHD, conduct disorder, autism spectrum disorder and tics

***Cannabis use is measured as cannabis use at age 15 or prior and used more than one time. It is based on self-reported data obtained at the 16-year follow-up.

****Missing data on 25 individuals on sociodemographic factors.

The mean Variance Inflation Factor value (VIF-value) for the multivariate analysis was 1.09 which indicates that the level of multicollinearity is acceptable.

The results found for the sensitivity analyses that view the outcome of SHM at age 16 as the number reported symptoms at age 16 on a continuous scale, can be seen in S2 Appendix. Cannabis use and psychotic experiences were both consistently significantly associated with age 16 hypomania across the unadjusted and adjusted univariate and multivariate analyses. We did not find a statistically significant linear association between SHM at age 11 and the number of SHM at age 16. Emotional disorders, neurodevelopmental disorders, insufficient sleep, and the interview-based measure of depressive symptoms were not associated with age 16 hypomania viewed on a continuous scale.

Moreover, the two sensitivity analyses of the multivariate analyses, without SHM and emotional disorders, respectively, did not markedly change the estimates (see S3 Appendix).

## Discussion

### Main findings

Any SHM symptom was clinician-rated in 5% of the children at age 11 years. As hypothesized, most of the examined clinical correlates at age 11, i.e. interview-based depressive symptoms, depressive disorders, neurodevelopmental disorders including ADHD, psychotic experiences, and insufficient sleep, were associated with SHM, except for the broader emotional disorders variable which mostly comprised anxiety disorders. As hypothesized, SHM at age 11 years was associated with self-reported SHM at age 16 years, also after adjustment for other putative risk factors, sex and socioeconomic factors. Furthermore, the multivariate analyses showed that particularly early psychotic experiences and cannabis use were robust risk factors of self-reported SHM at age 16 years.

### Strengths and limitations

Strengths of the study include the large study population, the comprehensive assessments of psychopathology in preadolescence, at age 11 years, and follow-up in early adolescence at age 16 years. We examined putative risk factors of SHM at a developmentally important age in preadolescence and early adolescence. Although the incidence rates of affective disorders, in particular depressive disorders, increase during adolescence, the early and mid-adolescence has so far been scarcely described in relation to the clinical correlates and precursors of hypomania.

The results should also be interpreted in the light of several limitations. Most importantly, SHM at age 16 years, was measured by self-report. Although the HCL-32 has been rather extensively used, the use of self-reported hypomania symptoms in the general population has been criticized for misclassification, i.e. including normal phenomena/behaviours as being hypomania, or simply tapping in to more general affective dysregulation not specific to hypomania/mania [15, 39]. Although we administered the HCL-32 as part of the face-to-face health examination and gave oral and written introduction to the experience of a hypomania-like episode with elevated mood, we cannot rule out the risk of over-reporting. However, the large proportion scoring 0 on the HCL-32 indicate that most respondents adhered to the instruction that hypomania symptoms should be considered within a context of a period with experiences unusual to them. The top 10% of the population endorsed having experienced no less than 20 out of 32 items of the HCL-32, suggesting a notable level of hypomania-like symptoms. Also, a small subset of individuals who may have experienced clinical episodes of hypomania/mania are expected to be part of the SHM-group in the current sample. We used a top 10^th^ percentile cut-off on the HCL-32 as our SHM outcome at age 16 years. An official cut-off on the HCL has not been developed, and our cut-off is arguably arbitrary. We chose the top 10^th^ percentile cut-off, because we expected that it would optimize the chance of identifying individuals who were likely to have had impairing mania/hypomania, without being too inclusive. Lastly, the HCL-32 includes question concerning both lifetime and present subclinical hypomania symptoms. This might have resulted in increased continuity of SHM. However, we expected that most of the adolescents would report recent symptoms whereas it was highly unlikely that symptoms experienced only in childhood would be reported only when answering the HCL-32 at age 16. Secondly, cannabis use was measured retrospectively at the 16-year follow-up, whereas the other putative risk factors were measured at the 11-year follow-up. This may have led to recall-bias and additionally caused a stronger association between cannabis use and SHM at age 16 years. Thirdly, the participants had been exposed to less psychosocial adversities compared to the non-participants. This selection might have affected the range of scores on clinical characteristics and thereby possibly attenuated the associations between the risk factors and the outcome of hypomania.

### Interpretation

SHM at age 11 years were associated with all the clinical correlates investigated at age 11 years besides a broad measure of emotional disorders. Particularly strong associations were found between SHM on one hand and interview-based depressive symptoms and depressive disorders on the other hand, in line with meta-analytic evidence [12], and as would be expected considering that depressive episodes are also part of BD in the course of illness [2]. The broader measure of emotional disorders, mostly comprising anxiety disorders, indicates that anxiety disorders were not associated with early SHM in preadolescence. Furthermore, we found an association between psychotic experiences and SHM in line with previous research [4, 20, 25, 26]. Although SHM and diagnosis of neurodevelopmental disorders, particularly ADHD, were associated in pre-adolescence, the longitudinal analyses did not suggest increased risk of SHM for youths with ADHD. The changing status of ADHD as clinical correlate to SHM could reflect an overlap of symptoms of SHM and ADHD in early age, whereas symptoms may gain greater specificity with age across adolescence. In line with our findings, a number of other community-based studies have found that ADHD in adolescence is only weakly associated with the development of hypomania in young adulthood [13], and not associated with later development of mania [17, 18].

In the longitudinal analyses, SHM at age 11 years was a precursor of self-reported SHM at age 16, also in the final adjusted models. This coincides with previous research on SHM in childhood, which has shown some stability throughout adolescence [8], which might help predict later BD [11]. Previous research has identified both depression [6] and anxiety as potential precursors of hypomania [46], yet they were relatively weakly associated with later self-reported SHM in the current cohort. This might in part be explained by the emotional problems and disorders often being transient or fluctuating in youths [5, 47]. A previous study in youths also found that emotional problems were not strongly associated with hypomania in young adulthood in the general population ALSPAC cohort [13]. Moreover, anxiety disorders early in life have been described as being non-specific regarding prediction of later psychopathology [48], which might explain the findings. On the contrary, anxiety in adolescence has also been found to be a significant risk factor of later BD in the general population [48].

Both psychotic experiences and cannabis use showed to be strongly associated with SHM in cross section as well as in the longitudinal analyses, in line with findings from other studies [4, 18, 20, 25, 26, 30, 49]. The finding that age 11 psychotic experiences were robustly associated with adolescent self-reported SHM can be interpreted in light of previous research describing psychotic experiences as a general marker of distress [50], and corroborates well with previous findings of the CCC2000, that psychotic experiences represent an underlying vulnerability of later psychopathology broadly [51]. The findings are also in line with BD and psychotic disorders sharing genetic- and environmental risk factors [21, 22], and moreover that mania can be viewed as part of psychotic symptomatology [19, 20]. The robust association between cannabis use and hypomania is consistent with previous findings from the ALSPAC cohort, showing that cannabis use in the adolescence increase the risk of developing hypomania in adulthood, and might be a viable target for prevention [18, 30].

The symptom reduced sleep is a part of hypomanic symptomatology, yet insufficient sleep was only associated with SHM in the cross-sectional analyses, and not in the longitudinal analyses. Insufficient sleep (i.e., less than 8.5 hours before weekdays) could be caused by many different factors, including gaming, use of social media, parental neglect regarding the supervision of bedtime etc., and most sleep patterns are probably fluctuating over the course of follow-up. In line with our findings, an association between sleep problems and hypomania in cross-section in a recent meta-analysis of community based studies [27].

Finally, in the sensitivity analyses the SHM at age 16 years were measured on a continuous scale. Although the overall findings from the main longitudinal analyses were replicated, the associations were somewhat less statistically robust. This might indicate that the self-reporting of low numbers of SHM symptoms at age 16 can be considered as a normal phenomenon, whereas scores at the extreme high end are more likely to be considered as potentially pathological and associated with other worrying psychopathology and behaviors.

In conclusion, the current study adds to the existing evidence that, SHM shows some continuity from preadolescence to adolescence. Additionally, psychotic experiences in preadolescence and cannabis use were strong predictors of later self-reported SHM, whereas the association between preadolescent emotional problems and disorders were less convincingly associated with SHM at age 16 years, and neurodevelopmental disorders were not. Thereby our findings indicate that early precursors can be identified and perhaps point to a window of opportunity for targeted prevention and early interventions, most obviously targeting early use of cannabis. The findings also underline that psychotic experiences in childhood are important to consider when screening for a wide array of later mental health problems. All longitudinal associations found in the current study should be considered in the light of SHM being self-reported with low face-validity at age 16. Our findings emphasize the need of a holistic and transdiagnostic approach when identifying the risk factors for later development of mental disorders [52].

## Supporting information

S1 Appendix HCL score distribution. (DOCX)

S2 Appendix Linear regression analyses. (DOCX)

S3 Appendix Sensitivity analyses. (DOCX)
