# Supplementary material for: Precursors of self-reported subclinical hypomania in adolescence: A longitudinal general population study
Source: PLoS One. 2021 Jun 18;16(6):e0253507. doi: 10.1371/journal.pone.0253507 (PMC8213158; doi:10.1371/journal.pone.0253507)
Supplement: S1 Appendix — (DOCX) [file pone.0253507.s001.docx]

**S1 Table. Hypomania Checklist 32 (HCL-32) score distribution.**

| *HCL-32 score* | *Frequency (%)* |
| --- | --- |
| 0 | 344 (38.52) |
| 1 | 3 (0.34) |
| 2 | 3 (0.34) |
| 3 | 5 (0.56) |
| 4 | 8 (0.90) |
| 5 | 7 (0.78) |
| 6 | 16 (1.79) |
| 7 | 7 (0.78) |
| 8 | 18 (2.02) |
| 9 | 16 (1.79) |
| 10 | 20 (2.24) |
| 11 | 19 (2.13) |
| 12 | 28 (3.14) |
| 13 | 39 (4.37) |
| 14 | 53 (5.94) |
| 15 | 59 (6.61) |
| 16 | 43 (4.82) |
| 17 | 34 (3.81) |
| 18 | 37 (4.14) |
| 19 | 43 (4.82) |
| 20 | 31 (3.47) |
| 21 | 19 (2.13) |
| 22 | 11 (1.23) |
| 23 | 9 (1.01) |
| 24 | 11 (1.23) |
| 25 | 5 (0.56) |
| 26 and above | 5 (0.56) |
