# Supplementary material for: Precursors of self-reported subclinical hypomania in adolescence: A longitudinal general population study
Source: PLoS One. 2021 Jun 18;16(6):e0253507. doi: 10.1371/journal.pone.0253507 (PMC8213158; doi:10.1371/journal.pone.0253507)
Supplement: S2 Appendix — (DOCX) [file pone.0253507.s002.docx]

S2 Appendix

**S4 Table. Univariate linear regression analyses - the association between symptoms/ diagnoses at age 11 and subclinical hypomania at age 16.**

| Symptoms/diagnoses at age 11 | subclinical Hypomania at age 16 Follow-up study population = 893 | |  |
| --- | --- | --- | --- |
|  | ***Crude analyses*** | ***Adjusted for sex and sociodemographic factors******* | ***Missing*** |
|  | **β (95% CI)** | **β (95% CI)** | **n** |
| Subclinical Hypomania | 0,10 (-0,002-0,24) | 0,10 (-0,001-0,25) | 0 |
| Emotional disorders* (ICD-10 diagnosis) | 0,05 (-0,01-0,14) | 0,05 (-0,02-0,14) | 4 |
| Interview-Based Depressive symptoms | 0,03 (-0,02-0,10) | 0,03 (-0,02-0,10) | 0 |
| Neurodevelopmental disorders** (ICD-10 diagnosis) | 0,003 (-0,06-0,10) | 0,0009 (-0,06-0,10) | 4 |
| Psychotic experiences | 0,12 (0,04-0,22) | 0,13 (0,05-0,23) | 0 |
| Insufficient sleep (≤8.5 hours) | 0,02 (-0,03-0,09) | 0,02 (-0,03-0,10) | 4 |
| Cannabis use*** | 0,13 (0,06-0,23) | 0,14 (0,06-0,23) | 27 |

* Emotional disorders include depression, anxiety and OCD, ** Neurodevelopmental disorders include ADHD, conduct disorder, autism spectrum disorder and tics, ***Cannabis use is measured as cannabis use at age 15 prior and used more than one time. It is based on self-reported data obtained at the 16-year follow-up. ****Missing data on 25 individuals on sociodemographic factors.

**S5 Table. Multivariate linear regression analysis – the association between symptoms/ diagnoses at age 11 and subclinical hypomania at age 16.**

| Symptoms/ diagnoses at age 11 | subclinical Hypomania at age 16 Follow-up study population, N = 893 | |
| --- | --- | --- |
|  | ***Crude analyses*** | ***Adjusted for sex (1= girls) and socioeconomic factors******* |
|  | **β (95% CI)** | **β (95% CI)** |
| SUBCLINICAL Hypomania | 0,09 (-0,02-0,24) | 0,09 (-0,02-0,24) |
| Emotional disorders* (ICD-10 diagnosis) | 0,06 (-0,02-0,17) | 0,06 (-0,03-0,16) |
| Neurodevelopmental disorders** (ICD-10 diagnosis) | -0,05 (-0,13-0,07) | -0,05 (-0,13-0,06) |
| Psychotic experiences | 0,09 (0,01-0,19) | 0,10 (0,02-0,20) |
| Insufficient sleep (≤8.5 hours) | 0,009 (-0,04-0,08) | 0,02 (-0,04-0,09) |
| Cannabis use*** | 0,17 (0,06-0,31) | 0,18 (0,07-0,33) |

* Emotional disorders include depression, anxiety and OCD, ** Neurodevelopmental disorders include ADHD, conduct disorder, autism spectrum disorder and tics, ***Cannabis use is measured as cannabis use at age 15 or prior and used more than one time. It is based on self-reported data obtained at the 16-year follow-up., ****Missing data on 25 individuals on sociodemographic factors.
