# Supplementary material for: Precursors of self-reported subclinical hypomania in adolescence: A longitudinal general population study
Source: PLoS One. 2021 Jun 18;16(6):e0253507. doi: 10.1371/journal.pone.0253507 (PMC8213158; doi:10.1371/journal.pone.0253507)
Supplement: S3 Appendix — (DOCX) [file pone.0253507.s003.docx]

**S3 Appendix. Sensitivity analyses**

**S1 Table. Multivariate analysis – the association between symptoms/ diagnoses at age 11 and subclinical hypomania at age 16 – not including emotional disorder.**

| Symptoms/diagnoses at age 11 | subclinical Hypomania at age 16 Follow-up study population, N = 893 | |
| --- | --- | --- |
|  | ***Crude analyses*** | ***Adjusted for sex (1= girls) and socioeconomic factors******* |
|  | **Relative Risk (95% CI)** | **Relative Risk (95% CI)** |
| SUBCLINICAL Hypomania | 1,80 (0,95-3,43) | 1,85 (0,98-3,49) |
| Neurodevelopmental disorders* (ICD-10 diagnosis) | 0,57 (0,23-1,40) | 0,55 (0,23-1,36) |
| Psychotic experiences | 2,13 (1,31-3,47) | 2,23 (1,34-3,62) |
| Insufficient sleep (≤8.5 hours) | 1,05 (0,63-1,77) | 1,16 (0,70-1,92) |
| Cannabis use** | 2,76 (1,67-4,55) | 3,04 (1,87-4,94) |

*Neurodevelopmental disorders include ADHD, conduct disorder, autism spectrum disorder and tics, **Cannabis use is measured as cannabis use at age 15 or prior and used more than one time. It is based on self-reported data obtained at the 16-year follow-up., ****Missing data on 25 individuals on sociodemographic factors.

**S2 Table. Multivariate analysis – the association between symptoms/ diagnoses at age 11 and subclinical hypomania at age 16 – not including subclinical hypomania at age 11.**

| Symptoms/diagnoses at age 11 | subclinical Hypomania at age 16 Follow-up study population, N = 893 | |
| --- | --- | --- |
|  | ***Crude analyses*** | ***Adjusted for sex (1= girls) and socioeconomic factors******* |
|  | **Relative Risk (95% CI)** | **Relative Risk (95% CI)** |
| Emotional disorders* (ICD-10 diagnosis) | 1,71 (0,96-3,05) | 1,74 (0,99-3,04) |
| Neurodevelopmental disorders** (ICD-10 diagnosis) | 0,51 (0,20-1,28) | 0,47 (0,19-1,19) |
| Psychotic experiences | 2,07 (1,27-3,38) | 2,13 (1,32-3,46) |
| Insufficient sleep (≤8.5 hours) | 1,12 (0,68-1,86) | 1,23 (0,75-2,01) |
| Cannabis use*** | 2,78 (0,68-4,58) | 3,09 (1,90-5,02) |

* Emotional disorders include depression, anxiety and OCD, ** Neurodevelopmental disorders include ADHD, conduct disorder, autism spectrum disorder and tics, ***Cannabis use is measured as cannabis use at age 15 or prior and used more than one time. It is based on self-reported data obtained at the 16-year follow-up., ****Missing data on 25 individuals on sociodemographic factors.
